# Enhancing Multimodal Learning Through Traditional Sporting Games: Marro360°

**DOI:** 10.3389/fpsyg.2020.01384

**Published:** 2020-07-07

**Authors:** Pere Lavega-Burgués, Rafael A. Luchoro-Parrilla, Jorge Serna, Cristòfol Salas-Santandreu, Pablo Aires-Araujo, Rosa Rodríguez-Arregi, Verónica Muñoz-Arroyave, Assumpta Ensenyat, Sabrine Damian-Silva, Leonardo Machado, Queralt Prat, Unai Sáez de Ocáriz, Aaron Rillo-Albert, David Martín-Martínez, Miguel Pic

**Affiliations:** ^1^Motor Action Research Group (GIAM), INDEST, National Institute of Physical Education of Catalonia (INEFC), University of Lleida, Lleida, Spain; ^2^Complex System Research Group, National Institute of Physical Education of Catalonia (INEFC), University of Lleida, Lleida, Spain; ^3^Motor Action Research Group (GIAM), National Institute of Physical Education of Catalonia (INEFC), University of Barcelona, Barcelona, Spain; ^4^Motor Action Research Group (GIAM), Institute of Sport, Tourism, and Service, South Ural State University, Chelyabinsk, Russia

**Keywords:** observational methodology, physical education, T-pattern analysis, motor conduct, motor praxiology, decision-making, motor interactions, physical effort

## Abstract

Different international organizations and initiatives highlight the contribution of the traditional sporting games (TSGs) to favor the diversity of knowledge, values, and attitudes necessary for today’s society. TSG such as Marro trigger multimodal learning contexts (driving conducts, interpersonal and organic relationships), with great interest in the educational and sports initiation field. The purpose of two studies presented in this manuscript was to examine the 360° multimodal strategic intervention (decisional, relational, and organic) of two teams faced in a Marro game. For this study, a quasi-experimental design was used composed by a single test applied to two non-equivalent teams. Mixed methods were used with an observational methodology in Quadrant III: nomothetic, punctual, and multidimensional. Fourteen university students participated [mean (M) = 20.49, standard deviation (SD) = 2.18]. Three internal logic variables were studied: outcome, role, and subrole; and three variables referred to the dimensions of motor conduct: relationship, risk in the decision, and physical effort. A mixed *ad hoc* registration system was designed with acceptable margins of data quality. For Study 1, cross-tabulations and classification trees were applied, while for Study 2 strategic T-patterns were identified. The relevance of the scoreboard (*p* < 0.001; *Effect Size* = 0.386) and the realization of the role (*p* < 0.001; *ES* = 0.091) for the study of multimodal strategic chains in the Marro game were confirmed. The detection of regularities in specific interaction (Hunters against Hares) by Theme (*p* < 0.005) allowed for interpretation of the process of strategic conducts of both teams during the game. Knowing the strategic chains of playful coexistence among equals through a multimodal range of variables and approaches has revealed an unusual dynamic picture. The study provides scientific evidence for the physical education teacher on the dynamics of the game of Marro. The pedagogical application of these contributions must be made according to curricular interests.

## Introduction

According to the Kazan action plan (UNESCO, 2017), TSGs are a fundamental intervention area for the acquisition of basic life skills; cognitive, social, and emotional skills; values; and attitudes that define socially responsible citizens. In addition, TSG are also important facilitators for sustainable development, inclusive education of cultural diversity, and peace.

The TSG testify to the local culture that has been transmitted over time, and their originality lies in the fact that they are social manifestations, which are expressed through motor action: body language. These are authentic cultural showcases that contain values and distinctive factors of their society; hence, UNESCO recognizes TSG as intangible cultural heritage (Parlebas, 2001).

Despite this, the TSGs have an insufficient presence in the university curricula and in the physical education classes, when compared to Olympic sports. Scientific evidences confirm the magnificent contribution of TSG to educate in values as necessary as respect, equality, peaceful coexistence, sustainability, diversity, and mutual help (Lavega et al., 2016).

The TSGs are based on a democratic pact, on a social contract (Rousseau, 1973; Parlebas, 2001). This is the first social lesson they give. In order to play a TSG, all participants should respect the rights and prohibitions established by rules. The observation of the application of the rules affirms that each game has an internal logic or identity card that tests its participants through different ways of relating to other participants, space, materials, and time (Parlebas, 2017a).

There is a large repertoire of TSG in which participants must interact with their partners and adversaries. These sociomotor games require the actors to have a constant dialogue with other people, whether they are members of the same team or rivals. Through these games, participants learn to enjoy the pleasure of meeting others (Lavega et al., 2014b; Muñoz et al., 2017). From the approach of social psychology, personal connections and group dynamics have also been studied (Graupensperger et al., 2019). The same sign of valence (Heider, 1946) used in TSG, guides the relationships of solidarity (positive valence) or social conflict (negative valence) (Böhm et al., 2018) within a given society. Although it should be noted that the conflict prism from TSG is an abstraction of a different nature, of great importance to act in the education of values (Lee, 1988; Bredemeier, 1991; Gibbons et al., 1995; Hernández-Mendo and Planchuelo-Medina, 2012).

The TSGs, like sports, are motor situations that have a system of rules and establish a competition between the participants. However, in the TSG, there is no presence of an institution (national or international federation). It is the players themselves who agree on the rules to be followed.

Under these conditions, each TSG has an internal logic. That is, it activates a different motor and social adventure, associated with original and varied rules according to the time and geography in which they are played. Therefore, they constitute an exuberant playful diversity (Parlebas, 2001) useful for physical education.

The learning caused by TSG is aimed at acquiring the motor competence that offers testimony through motor conducts of knowing, knowing how to do, knowing how to be, and knowing how to act (Parlebas, 2017a). That is, one learns to agree, to cooperate, and to respect others through motor action. For this reason, participants find themselves in real, not imaginary, scenarios of contextual learning that allow the different dimensions of their personality to be put into action. It is about multimodal learning contexts (Ward et al., 2017). Within each motor action (a pass, a displacement, a jump) can be found a physical effort (organic dimension), decision (cognitive dimension), emotion (affective dimension), and communication (relational dimension).

For each person, this multimodal unitary intervention (which we could call 360°) is different. These are motor conducts in which the external meaning (the observable part of motor execution) and the internal meaning that the person gives to his motor intervention are linked (Parlebas, 2001).

The research by Brasó and Torrebadella (2017) indicates that Marro is a game with possible antecedents in ancient Greece, known in many European countries with different denominations: Barres or Le jour e la nuit in France, Prisoner’s Bars or Prisoner’s Base in England, Giorno and notte, Paladini, Barrierre, or Barre in Italy, Tag und nacht or Das Mattmachen in Prussia and Germany, and Marro, Regate, Hurto del cuerpo, Rescat, or Riscat in Spain. These authors affirm that throughout history, this game has been practiced by people of different ages and social classes. It reached its full popularity in the fifteenth and sixteenth centuries among young people in rural and school contexts. Its incorporation into the school is very clear from the seventeenth century. Later, in the nineteenth century, this game, same as other TSGs, followed a process of pedagogization and school institutionalization.

### Marro Game Rules

Although it can be played in different ways, in this case, two teams with the same number of players, placed in a protected area (Home) behind a line at one end of a rectangular field, face each other. Each player who leaves Home may chase and capture (as a Hunter) all opposing players (playing as Hares) who have left before him/her. In these circumstances, a player can play the role of Hunter before his/her Hares. However, they should know that if an adversary leaves his/her Home after him/her, the latter will have “Marro” on him/her and will become a Hunter, so that he/she will become a Hare. In that case, the person must decide whether to continue chasing any of his/her Hares or flee from his/her Hunter.

When a player catches a Hare, he/she takes it to the Prisoner area, on a side 1.5 m away where it will be placed forming a chain (holding hands) with the rest of his/her team’s Prisoners. If a player away from Home (Hunter or Hare) manages to touch a Prisoner of the chain, all those who are forming the chain are free, although they can be captured again by any adversary before returning Home. In some ways of playing, the team that first captures all opponents or the team that, after a game time (e.g., 8 min), has the highest number of captured Prisoners from the opposing team wins.

### The Internal Logic of Marro Encourages Decision Motor Conducts

While knowing the code of rules of the game is necessary, it is not enough to reveal the regularities that the Marro game contains. Although each player makes their own decisions, there is an underlying order that is the same for all actors, regardless of their age, gender, or cultural background.

The theory of motor action or motor praxiology (Parlebas, 2005) has universal or operational models that represent the basic structures (Levi-Strauss, 1963) of the operation of a game. There are seven universals that reveal the underlying order that contains the internal logic of any game. To study decision-making, two key universals are identified: the network of changes of sociomotor roles and the network of changes of sociomotor subroles.

#### The Network of Changes of Sociomotor Roles

Through this model the importance of appropriate decision-making in the game of Marro could be identified. A role corresponds to the potential motor conducts referred to as the limitations, rights, and prohibitions prescribed for one or more players by the rules of the game (Parlebas, 2001). In the Marro, there are three roles: Home (being in the protected area), Field Player (alive), and Prisoner.

Unlike team sports, players’ decisions are conditioned by an excellent management of the relationship with time, since the “moment” of leaving creates the possibility of having Marro (being a Hunter) or receiving Marro (being a Hare) over rivals. In addition, it may be that a player is potentially a Hunter (for a rival Hare) and a Hare (for a rival Hunter who has left afterward) at the same time. In these circumstances, players will decide what role they will play in each game sequence.

The systematic observation of the game has allowed us to identify three strategic roles associated with the role of a live player:

– Hunter: player who has Marro on opponents who have left “before” Home.
– Hare: player who has left Home “before” one or more opponents.
– Neutral: player whose decisions do not have an intention directed to the other participants.
  Through observation, it also identifies another strategic role in situations of disagreement:
– In Conflict: when two or more players stop playing to discuss any disagreement during any sequence of actions they have shared.

The dynamism of the game is associated with the transition from one role to another, depending on the choices each player makes. Representation based on graph theory (Berge, 1958) identifies roles through points and role changes through lines. Loops or lines on the same role show that given a possible change from one role to another, some players may remain in that same role.

#### The Network of Changes of Sociomotor Subroles

Each role contains different subroles considered as the minimum unit of action loaded with strategic significance.

The internal logic predetermines the possible changes between subroles allowing each player, according to their strategy, to make different decisions corresponding to the transition between different subroles. This, in turn, involves the transition between different roles. The systematic observation of this game has identified the following subroles:

Home (Hom)

– On hold (CEE): Player who is in the Home zone in a passive attitude: standing, with crossed arms, with no intention of leaving.
– On alert (CEA): Player who is in the Home zone in an active attitude, moving, running, feinting.
– Leaving (CSL): Player leaving the Home zone.

Hunter (Hun)

– Menacing (ZAM): Hunter who stands (without moving) but in an active attitude, feinting, ready to attack.
– Tracker (ZPS): Hunter who is chasing (running).
– Catcher (ZCT): Hunter that is at the moment of capturing.
– Conveyor (ZTL): Hunter who is moving his/her victim to the Prisoners’ area.
– Go to save (ZIS): Hunter who is in the process of going to save but is not doing it yet.
– Rescuer (ZSV): Hunter who is currently saving his/her fellow Prisoners.

Hare (Har):

– On alert (LEA): Hare that stands (without moving) but in an active attitude, feinting, prepared to avoid being attacked.
– Provoker (LPV): Hare that is catching the attention of Hunters. Fundamentally on the first play of the game.
– Runaway (LHD): Hare escaping persecution or the threat of Hunters.
– Protector (LPT): Hare that protects his/her fellow Prisoners and commits suicide.
– Enter Home (LEC): Hare that passes from the living area to the Home area.
– Rescuer (LSV): Hare that, in his/her flight from the Hunter, goes to the Prisoners’ area of his/her team to try to save them.

Neutral (N)

– On hold (NEE): Neutral player (in the living area) that is wandering, standing, or moving, waiting to make another more influential decision in the game.
– Returning Home without threat (NRG): Neutral player (mainly Hares) who is returning Home quietly because he/she no longer feels a threat from Hunters. That return can be walking or running.

Prisoner (Pri)

– Go to prison (PIP): Hare that is captured and moved to the prison of the rival team.
– On hold (PEE): Prisoner who waits, with whatever attitude, to be released to adopt a new role in the game.

Conflict (F)

– In conflict (EC): Players who, for whatever reason, do not play. The stoppage of the game can be partial (players who are in conflict, but the rest are still playing) or total (the entire game is paralyzed). Whether it is total or partial, it will be categorized “in conflict” until the moment the game is restarted with the motor decision that is taking place at that moment of restarting the game.

Adopting a subrole involves deciding a level of risk in the intervention. Considering the consequences of being able to be captured or not, the subroles can be classified as (see Table 1): conservative (passive and risk-free decisions), risky (can be captured), and neutral (in the live role it is an active decision (e.g., back Home); in the Prisoner role, it means not having an alternative choice).

**TABLE 1:** Relation of roles, subroles, and their level of risk and decisional relationships.

Roles Rules	Strategic Roles		Sociomotor Subroles				
Name	Name	Cod	Name	Code	#	Risk decision-making	Type relational decision-making
Home	Home	1	On hold	CEE	1	1 Conservative	3 Neutral
			On alert	CEA	2	1 Conservative	3 Neutral
			Leaving	CSL	3	2 Neutral	3 Neutral
Alive	Hunter	2	Menacing	ZAM	4	3 Risky	2 Opponent
			Tracker	ZPS	5	3 Risky	2 Opponent
			Catcher	ZCT	6	3 Risky o	2 Opponent
			Conveyor	ZTL	7	2 Neutral	2 Opponent
			Go to save	ZIS	8	3 Risky	1 Partner
			Rescuer	ZSV	9	3 Risky	1 Partner
	Hare	3	On alert	LEA	10	1 Conservative	3 Neutral
			Provoker	LPV	11	3 Risky	2 Opponent
			Runaway	LHD	12	3 Risky	2 Opponent
			Protector	LPT	13	3 Risky	1 Partner
			Enter home	LEC	14	2 Neutral	3 Neutral
			Rescuer	LSV	15	3 Risky	1 Partner
	Neutral	4	On Hold	NEE	16	1 Conservative	3 Neutral
			Returning home without threat	NRG	17	2 Neutral	3 Neutral
Prisoner	Prisoner	5	Go to prion	PIP	18	2 Neutral	3 Neutral
			On hold	PEE	19	2 Neutral	3 Neutral
	Conflict	6	In conflict	EC	20	2 Neutral	4 Conflict

### The Internal Logic of Marro Encourages Conducts of Interpersonal Relationship

The Marro game is a miniature society (Parlebas, 2001) in which players share interpersonal relationships. The relational order of the game can be revealed by two universals:

*The motor communication network* reveals the underlying relational structure (Parlebas, 2002), that is, the type of motor relationships that are to be activated.

Marro players are related through two options of motor interaction (Parlebas, 2002): (a) motor communication or positive communication corresponding to a transmission motor relationship explicitly provided by the rule and which, in the case of Marro, occurs with the transmission of a positive sociomotor role (touching fellow Prisoners to release them); and (b) the countercommunication or negative communication referred to as motor interrelation of opposition between adversaries and which, in the case of Marro, corresponds to the transmission of a negative role (touching an opponent to turn him/her into a Prisoner).

**FIGURE 1:**
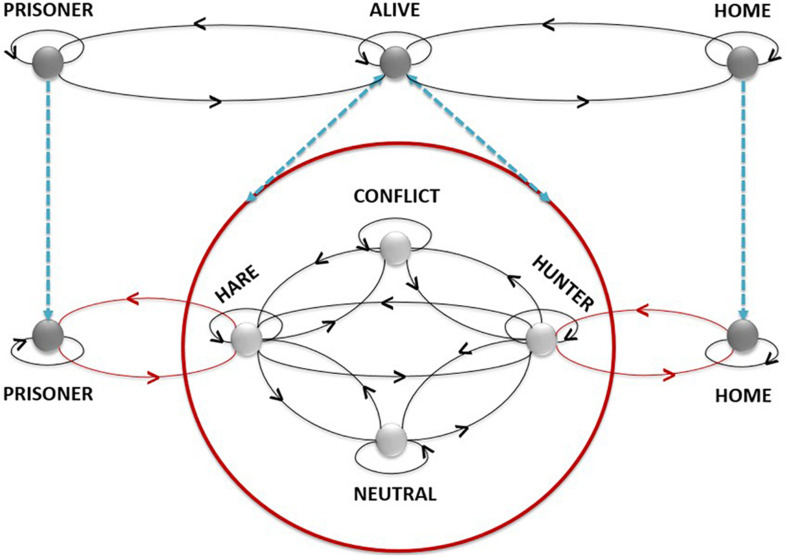
Network of sociomotor role changes in Marro.

**FIGURE 2:**
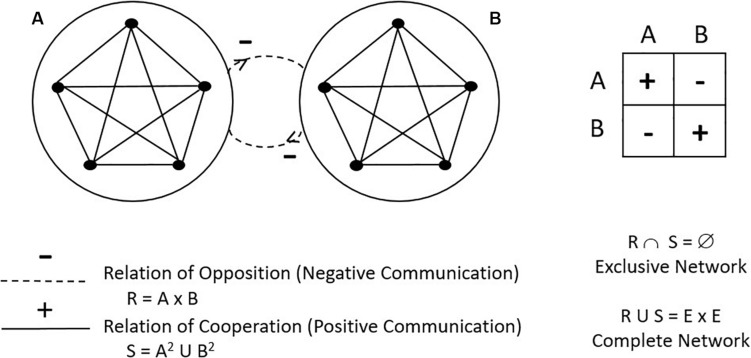
The motor communication network in Marro game.

The motor communication network represents players through points (Figure 2) by roles (Figure 1). The continuous lines that unite them show the relationship of cooperation (solidarity) between the players, and the discontinuous ones correspond to the relations of opposition (rivalry). The Marro game corresponds to a team duel (two “collective actors” who describe a zero sum, according to game theory, since what is won on the one hand is lost on the other), symmetrical (equal number of players and roles), exclusive (each player can only be a companion or adversary of the other participants), and stable (remains on the same team during the entire game). The network is complete because each pair of vertices is connected to an edge of positive or negative relationship (Parlebas, 2002).

*Goal interaction network* is a subset of the motor communications network because it only examines the interactions that “outcome” success or failure in the game (Parlebas, 2001). In the case of Marro, each success is associated with capturing an opponent (each Prisoner involves adding a point, opposition relationship) and also releasing the partners (it implies leaving the rival’s outcome to zero, cooperative relationship). Unlike sports, whose brand interaction network is of opposition – since they only score points through rivalry relationships (scoring goal or basket on the opposite zone) – the Marro game retains a mixed goal interaction network: success is achieved by looking for both partners and opponents.

The twenty subroles or possible decisions of the Marro game have also been grouped according to whether these subroles indicate a relation with partners or with adversaries. Observation has identified neutral relationships (being at Home, being in prison) and conflict (when discussing a disagreement) (Table 1).

### The Internal Logic of Marro Causes Motor Conducts of Different Physical Effort Intensity

This game corresponds to a team duel in which the intervention of any player is conditioned by a constant decision-making during the development of the game. As in other games or team sports, the other participants carry out messages associated with unforeseen events, an informational uncertainty that translates into an acyclic and intermittent strategic intervention. In these conditions, players must maintain different intensities of physical effort with intermittent motor actions of high intensity interspersed with moments of recovery and pause (Bangsbo, 2000). In addition, that intensity can change according to the outcome (score), the team’s strategy, and the decision-making of each player, as well as their possibilities, their way of understanding the internal logic of the game, and their sports history or motivation (Apostolidis et al., 2003).

### The Need to Go Further

The review of the specialized literature confirms that the investigation of traditional games in educational contexts is scarce compared to the proliferation of studies on sport (cf. Navarro and Triguero, 2009). The motor praxiology is a discipline that focuses the attention on TSG as an object of study and that has generated a considerable amount of research on the effects of the TSG on emotional, relational, cognitive well-being, and decision-making of the protagonists (cf. Lavega et al., 2016).

From the observational methodology, studies focusing on traditional games have been essentially based on one or two dimensions of study, using different statistical strategies (generalized linear models, classification trees) (i.e., Lavega et al., 2014a). As far as we know, no previous study has been performed in order to interpret with a rigorous methodology an integrated view of the intervention of players in a TSG from a systemic point of view: decisional, relational, energetic, and various analysis approaches, that is, a 360° approach.

In the present study, this approach is a requirement imposed to propose a new and unknown integral approach for the study of team strategies in games or in team sports. The lack of approaches based on T-pattern analysis or predictive models to identify hidden strategic regularities is a new challenge. Therefore, it is about trying to reveal part of the interactive process that occurs during a game and facilitate the verification of evidence to be applied in the educational field or sports initiation.

Based on this theoretical framework, the article focuses its interest on identifying the 360° multimodal strategic development processes (decisional, relational, and organic) of two teams that play under the regulatory framework of the Marro game.

## Materials and Methods

### Design

The study followed an associative strategy (exploring the functional relationship between variables) and corresponded to a comparative predictive design based on group comparison (Ato et al., 2013). For this study, a quasi-experimental design was used composed by a single test applied to two non-equivalent teams. The study design (observational methodology) was nomothetic, punctual, and multidimensional (Anguera et al., 2011). It was nomothetic, because two collective units (teams) composed of players were studied. When a single concrete action was performed, it was considered punctual. A mixed system of field format and E/ME category system was used. Therefore, it would be a multimethod study (Teddie and Tashakkori, 2010; Anguera et al., 2014; Anguera et al., 2018).

### Participants

In this study, 14 players (seven per team) participated (10 male and 4 female, age range = 18–26 years, M*_*age*_* = 20.49 years, SD = 2.18) of the first course of physical activity and sport sciences at the University of Lleida, enrolled in the Theory and Practice of Motor Game. The distribution between women and men maintained the same proportion as in the class group, with a predominance of the masculine gender over the feminine one. The studied participants who developed a game of Marro were chosen at random. This reduced number of players is justified by the large amount of data (number of observations) recorded and analyzed during 480 s. The sum of 480 s accumulated by each player in interaction with their peers and rivals draws the performance, for each variable, within their respective team.

All participants signed an express authorization authorizing their filming (Declaration of Helsinki). In addition, the study was review and approved by the Ethics Committee for Clinical Research of the Catalan Sports Council [07/2019/CEICEGG].

### Procedure and Materials

Six variables were used in the study. The relationships between variables and categories are described below. Three variables corresponding to the internal logic of the game:

(i) “Dynamic Score (Outcome).” The following coding was used for the different outcomes (scores): draw (ZE), +1 (ON), +2 (TW), +3 (TH), +4 (FO), +5 (FI), -1 (NO), -2 (NT), -3 (OH), -4 (NFO), -5 (NFI).
(ii) “Role”: understood as categories of rights and prohibitions that players have that allow them to take subroles or minimum decision units (Table 1). Of the six possible decision scenarios: strategic roles (HUN), Hare (HAR), Home (HOM), Prisoner (PRI), in conflict and neutral, special attention was paid to the roles of Hunter and Hare, since they originate different strategic options in the different dimensions: cognitive (subrole), decisional risk (conservative, neutral, and risky), relational (looking for a partner or adversary), and organic (sedentary, light, moderate, and vigorous).
(iii) Subrole decision. Twenty decision subroles were identified (see Table 1).

Three variables linked to the dimensions of motor conducts:

(i) “Relation.” N: neutral, when ambivalent or indistinct (N); relationship with the opponent (A); relationship with the partner (C); and when there is a conflict of interest between the players (X).
(ii) “Decision Risk.” Risky decisions (RI), neutral decisions (NE), and conservative decisions (CO).
(iii) “Energy” is an indicator of the intensity of physical effort made by the players based on the data extracted from the accelerometer record. The cut-off points proposed by Troiano et al. (2008) to categorize the energy variable in effor t intensity: Sedentary (S): 0–2 CPS (No. of frequencies per second); Light (L): > 2–34 CPS; Moderate (M): > 34 to 100 CPS; Vigorous (V): > 100 CPS.

#### Phase 1: Playing in Practice and Authorizations

The Marro game was carried out by the regular teacher of the area during his regular class schedule, in a 42 m × 25 m artificial grass outdoor sports court.

Since none of the participants knew Marro, the game was explained in a previous class, it was put into practice for several minutes to favor its understanding, and tests were made for its filming at the same time as doubts raised by the players were solved. Once all doubts were dispelled, participants were invited to play after 2 days in this experience. Before starting the game, the participants performed a series of exercises adapting to the intensity of the physical effort of the game.

To make the recording, two Sony DCR-SX21 model cameras located at both ends of the game track were used. The recording time was continuous, without interruptions, until completing about 480 s. All shots were recorded far enough to ensure the anonymity of the players.

Triaxial accelerometers (ActiGraph GT3X + accelerometer; ActiGraph LLC, Pensacola, FL, United States) were used to record the intensity of the effort. The accelerometers were placed laterally at the waist of the players and fastened with an elastic band. Accelerometers were programmed to record movement at a frequency of 60 Hz.

#### Phase 2: Registration Tool and Data Quality

For the analysis of the data from the filming, an *ad hoc* registration tool was designed with exhaustive and mutually exclusive categories (Chacón-Moscoso et al., 2019). This allowed the use of ludograms (Parlebas, 2001) to record the driving strategy of the players in the different dimensions, corresponding with: sequence of roles and subroles, decisional risk, relationships, and physical effort assumed by a player in each second during game development.

The mixed system created for this purpose was used because it takes advantage of a field format and a category system. On the other hand, there was the flexibility of the field format adapted to new and unexpected playful events of the game, from categories initially identified deductively, according to the theoretical bases used. Categories were also exploratory or inductively identified as a result of the observation made by the observer’s team. The rigor of the category system was guaranteed by relying on the theoretical foundation of the science of motor action (Parlebas, 2001, 2017a).

The data were sequential (Chacón-Moscoso et al., 2019) since in each observation there could only be a single category. The type of parameters was secondary since the data were derived from a registry of primary indicators (role, subrole, and energy) that subsequently gave rise to different categories of risk, relationship, and energy intensity.

To address the quality of the data, different methodological strategies were followed. First, the observers had at least 2 years of experience in the observational methodology and its application. All of them were members of the GIAM research group, interested in the motor action. The observation tool was described and agreed by GIAM following the subroles (categories) and roles (criteria) of the Marro game.

After using the registration tool, it was implemented with different modifications and improvements, in order to ensure the quality of subsequent registrations.

The monitoring game action made by the observers was focal, that is, player by player. Thus, an independent record of each player was obtained. When the mixed system of consensual and definitive registration was reached, an observer manual was prepared, describing the categories (determination of roles and subroles) with the respective degree of freedom of the categories.

Next, although five observers were trained, just two of them were selected to code all players from both teams for the whole duration of the game, intra- (coder 1_coder 1) and interobserver (coder 1_coder 2) reliability by applying the Generalizability Theory (Cronbach et al., 1972; Ysewijn, 1996; Cardinet et al., 2010; Blanco-Villaseñor et al., 2014; Hernández-Mendo et al., 2016).

A match of the Marro game in which 14 players participated was analyzed: Two teams of seven players each and a duration of the game of 8 min. The results showed that the variability of the instrument was associated with the categories (93.7%) and with the categories/observer interaction (6.3%). The overall analysis of the relative and absolute G coefficient (0.98) revealed that the accuracy of the results was optimal.

#### Phase 3: Preparation of Two Databases for Analysis

All observed events are associated with a series of seconds between which that variable or that set of variables is activated in each player. Thus, the union of these players in their respective organizations forms the team unit.

The data collection had two procedures:

The observational procedure, carried out by a team of five observers allowed the recording of data of the variables outcome, role/subrole, risk in the decision and relation from the visualization of the videos, and the second-to-second record of the ludic events developed by the players.

The data recorded by the accelerometers (energy variable) were downloaded and analyzed using the ActiLife 6.0 software (ActiGraph, Pensacola, FL, United States). The data were integrated in periods of 1 s obtaining 480 s per participant. The intensity of the effort expressed quantitatively by means of the magnitude vector in counts per second was transformed into a categorical variable using the cut-off points indicated above.

Each player intervened for 480 s, coinciding with the number of rows in Microsoft Office v.2010 Excel, also used to transform the magnitude of movement variable (quantitative) into categorical (sedentary, light, moderate, or vigorous).

In order to carry out Study 2, the database was prepared to apply the package Theme (2017) software, with the aim of detecting T-patterns strategic Marro360°chains. Thus, those sequences of repeated variables were eliminated so that, on the one hand, the database became lighter and faster to analyze and, on the other, greater dynamics of the variables were achieved.

Through the programming language Phyton v 3.7, a script or command file was designed to eliminate the sequences of repeated variables (more than once in a row). That is, each time a type combination appeared (ZE, APS, A, RI, M) it was included for the analysis, but if this same combination was repeated next, then it was eliminated. Thus, the cumulative duration (number of seconds) of the sequences was available, and type IV data were obtained (Sackett, 1978). This procedure was applied exclusively for T-pattern analysis. It is of crucial importance to recognize that the internal logic of the game establishes a dynamic of action in which leaving before or after the adversary identifies a “when” of the playful event, with direct impact on the outcome (capturing an adversary or saving a Prisoner). This temporal dynamic guides the driving strategies of the social fabric that is the Marro game.

#### Phase 4: Data analysis

##### Phase 4.1: Data analysis in study 1

This study corresponds to a mixed methods design (Chacón-Moscoso et al., 2019). Using the IBM SPSS Statistics, v. 25 (2017) statistics analysis tool. Cross-tabulations (Pearson’s Chi-square test) were carried out with special attention to adjusted residuals (ARs) > 1.96 or < –1.96 (Gómez et al., 2019), and decision trees. For both analyses, levels of statistical significance were started (*p* < 0.05). The effect sizes were calculated using the Cramer’s V test. The interpretation was based on: 0.10 = small effect, 0.30 = medium effect, and 0.50 = large effect (Cohen, 1988). In order to elaborate the most representative figures of the Hunter and Hare roles, the sets of variables equal to or greater than 2% in the blue and red teams were selected. This selection was represented exclusively in Figures 7, 8, with a total of 1,072 occurrences (70.4%) in both roles and teams.

To determine the interaction between the variables, the multivariate QUEST classification technique (Quick, Unbiased, Efficient, Statistical Tree) was used. This was a fast-running binary procedure compared to other models already used in TSGs (Pic et al., 2019) to execute the ramifications. The tree used was due to a supervised learning algorithm, used in artificial intelligence to know the predictive capacity of the model when analyzing the performance of both teams.

The following requirements were assumed for the construction of the QUEST model: (i) a restriction of five levels of maximum tree depth, (ii) minimum cases (=100) in parent Node, and minimum cases (=50) in child Node, (iii) significance level for splitting nodes (*p* < 0.05); and (iv) the validation was approached by means of split sample, being used (randomized) 80% of the cases for the training, and 20% in the final test.

##### Phase 4.2: Data analysis in study 2

Following the mixed methods (Chacón-Moscoso et al., 2019) and a quasi-experimental approach, the implications of the temporal dimension in the Marro game, together with the ambition to apply a 360° approach of whole variables, made the use of a multivariate technique known by THEME (Casarrubea et al., 2015, 2019a, Aiello et al., 2020) pertinent. THEME is an algorithm that reveals temporal regularities, selected when examining the chains of variables, including in the analyses of the temporal distribution of events. Therefore, according to Magnusson (2000, p. 94–95) “if A is an earlier and B a later component of the same recurring T-pattern, then, after an occurrence of A at t, there is an interval [t + d1, t + d2] (d2 ≥ d1 ≥ d0) that tends to contain at least one occurrence of B more often than would be expected by chance.” The search parameters of T-patterns included for the assessments were significance levels (*p* < 0.005) and a minimum of four occurrences. A comparison was made between the results obtained (real data) with their randomization to validate the analyzes proposed (Brill and Schwab, 2019).

## Results

### Results: Study 1

#### The Outcome (Score) of Both Teams

Differences were found in their respective outcomes (*p* < 0.001; *ES* = 0.386). Both teams shared most of the game the ZE(0) draw outcome (blue and red: *n* = 1,288; 19.2%). Blue was mainly with ON (+ 1) outcome (*n* = 672; AR = 5.9; 10%) and NT (–2) (*n* = 504; AR = 15.1; 7.5%). The red team participated mostly with the score NO (–1) (*n* = 672; AR = 5.9; 10%) and TW (+ 2) (*n* = 504; AR = 15.1; 7.5%). The TH (+3) outcome was developed exclusively by the red team (*n* = 266; AR = 15.1; 4%).

#### Comparison of Outcomes Exclusively in the Hunter and Hare Roles

Significant differences were found when selecting two determining roles in Marro game (*p* < 0.001; *ES* = 0.353).

The red team obtained a higher number of records (*n* = 841; 55.3%) than the blue team (*n* = 681; 44.7%). The greatest differences in order, following the AR, were under an NT (–2) outcome in blue team (*n* = 104; AR = 7.2; 6.8%) and red team (*n* = 38; AR = –7.2; 2.5%).

In the second position, with a TW (+ 2) outcome the red team registered the following values (*n* = 134; AR = 6.9; 8.8%), different from the blue team (*n* = 33; AR = –6.9; 2.2%). Similar ARs were found OH (–3) exclusively in the blue team (*n* = 37; AR = 6.8; 2.4%) versus the red team (*n* = 0; AR = –6.8; 2.4%). The outcomes were favorable to the blue team, following the AR in ON (+ 1) (AR = 2.0) and ZE (0) (AR = 1.5) while higher outcomes by the red team were observed in NO (–1) (AR = 3.4) and TH (+ 3) (AR = 6.5).

##### Roles in both teams

The Marro’s game lasted for 6,720 s (resulting from the intervention of 14 players during 8 min). All observation periods were concatenated, player by player. Based on the characterization offered through the six strategic roles (*p* < 0.001; *ES* = 0.091) (Home, Hunter, Hare, Prisoner, Conflict, and Neutral), the two teams were mostly at Home (*n* = 2669″; 39.7%; red *n* = 1340″; AR = 0.7; 20.1% and blue *n* = 1320″; AR = –0.7; 19.6%), then Prisoner (*n* = 1197″; 17.8%; (blue *n* = 693″; AR = 6; 10.3% and red *n* = 504″; AR = –6; 7.5%), neutral (*n* = 1103″; 16.4%; (blue *n* = 571″; AR = 1.3; 8.5% and red *n* = 532″; AR = –1.3; 7.9%).

When attention was paid to the Hunter and Hare roles it was found that the intervention in the Hunter (HU; *n* = 970″; 14.42%) and Hare (HA; *n* = 522″; 8.23%) roles was developed during 1,522 s (22.65%). Significant differences were found in the intervention of the two teams in the role of Hunter and Hare. The red team spent more time in the role of Hunter (red *n* = 526″; AR = 2.9; 7.8% and blue *n* = 444″; AR = –2.9; 6.6%) and also in the role of Hare (red *n* = 315″; AR = 3.5; 4.7% and blue *n* = 237″; AR = –3.5; 3.5%).

##### Subroles in both teams

The game mainly showed decisions at Home (CEA, on alert *n* = 2,355″; 35%), Prisoner on hold (PEE, *n* = 906″; 13.5%), Hunter chasing (ZPS *n* = 631″; 9.4%), Neutral on hold (SEN *n* = 575; 8.6%), Returning Home without threat (NRG *n* = 528″; 7.9%) and Hare fleeing (LHD *n* = 423″; 6.3%). Finally, Prisoner go to prison (PIP *n* = 291″; 4.3%). Subsequently, in conflict (EC *n* = 229″; 3.4%), at Home (CSL leaving *n* = 221″; 3.3%), Hunter conveyor (ZTL *n* = 162″; 2.4%), at Home (CEE on hold *n* = 93″; 1.4%), and Hare on alert (LEA *n* = 74″; 1.1%).

Differences were found between the subroles of the Hunter-Hare roles (*p* < 0.001; *ES* = 0.155) of both teams. The subroles over 5% of the total and two ARs (positive or negative) were selected. Thus, the most used subroles were ZPS (*n* = 631″; 41.5%), with lower frequencies the blue team (*n* = 290″; AR = 0.8; 19.1%) than the red team (*n* = 341″; AR = –0.8; 22.4%).

The red team (*n* = 238″; AR = 0.5; 15.6%) exceeded the blue team (*n* = 185″; AR = –0.5; 12.2%) in LHD (27.8%) and similar records in both teams using ZTL (10.6%) were found in the red team (*n* = 82″; AR = –1.3; 5.4%) and blue team (*n* = 80″; AR = 1.3; 5.3%). In the last subroles selected by percentage in both ZAM teams (*n* = 77″; 5.1%), being in red (*n* = 37″; AR = –1.3; 2.4%) was similar to the blue team (*n* = 40″; AR = 1.3; 2.6%). Based on the differences, in terms of AR, in LPV (*n* = 19″; 1.2%) the red team exceeded (*n* = 19″; AR = 3.9; 1.2%) the blue team (*n* = 0″; AR = –1.2; 0%). However, the results were reversed in LPV since the blue team (*n* = 24″; AR = 2.7; 1.6%) exceeded the red team (*n* = 12″; AR = –2.7; 0.8%).

Still regarding the Hunter and Hare roles, no especially relevant differences were found when examining the level of risk of the players’ decisions (*p* < 0.068; *ES* = 0.59). Although it could be reported that more neutral decisions were found in the blue team (*n* = 104″; AR = 2.1; 6.8%) than in the red team *(n* = 98″; AR = –2.1; 6.4%).

##### Motor relationships in both teams

Statistically, the teams were different (*p* < 0.001; *ES* = 0.070) although both mainly showed neutral relations (*n* = 5,079″; 75.6%). Subsequently, the relations were oriented toward the adversary (*n* = 1,344″; 20.0%). There were few conflicting relationships (*n* = 229″; 3.4%). Relationships toward team partners were very scarce (*n* = 68″; 1.0%).

In neutral relations, the blue team surpassed the red team (blue *n* = 2,636″; AR = 5.5; 39.4%; red *n* = 2443″; AR = –5.5; 36.4%). The red team showed more relations with the adversary (blue = 605; AR = 4.1; red = 739 AR = –4.1), more conflict relations (blue *n* = 95; AR = —2.6; 1.4%; red *n* = 134; AR = 2.6 2.0%), and also more relationships with teammates (blue *n* = 24; AR = –2.4; 0.4%; red *n* = 44; AR = 2.4; 0.7%).

By segmenting records with Hunter and Hare roles exclusively, no significant effects could be found (*p* = 0.249; *ES* = 0.043), and the differences disappeared.

##### Physical effort in both teams

Based on all roles, the following statistical relevance was found (*p* < 0.001; *ES* = 0.051). The participation in the Marro game involved a mostly moderate energy (*n* = 2,302″; 34.3% of the time recorded) and also vigorous (*n* = 1,957″; 29.1%). Sedentary (*n* = 1,333″; 19.8%) and light interventions (*n* = 1,128″; 16.8%) reached lower values. The two teams showed similar sedentary conduct values (blue: *n* = 672″; AR = 0.3; 10.00%; red: *n* = 661″; AR = –0.3; 9.8%).

The blue team showed a mostly moderate conduct (blue: *n* = 1,219; AR = 3.5; 18.10%; red: *n* = 1,083″; AR = –3.5; 16.1%). The red team was more vigorous (blue: *n* = 952″; AR = 1.4; 14.2%; red: *n* = 1005″; AR = –1.4; 15.0%) and light (blue: *n* = 517″; AR = –3.1; 7.7%; red: *n* = 611″; AR = 3.1; 9.1%).

Exclusively in the Hunter and Hare roles, there were no significant differences in the level of effort between teams (*p* = 0.359; *ES* = 0.046).

##### Predictive capacity of variables on the conduct of both teams

Through the decision tree, the predictive capacity of the internal logic variables (Outcome, Role) and the different dimensions of the participants motor conducts (risk in the decision, type of relationship, level of physical effort) were studied (Figure 4).

**FIGURE 3:**
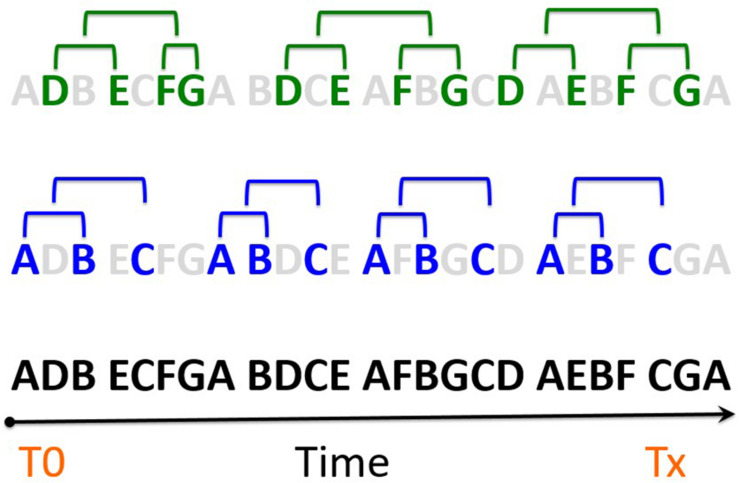
Temporary events and regularities in a sequence of game (taken from Casarrubea et al., 2015). The elimination of superfluous events on a timeline is the procedure applied in Figures 5, 6A,B. The bottom-line disorder hides a specific order based on events and time distances (top line).

**FIGURE 4:**
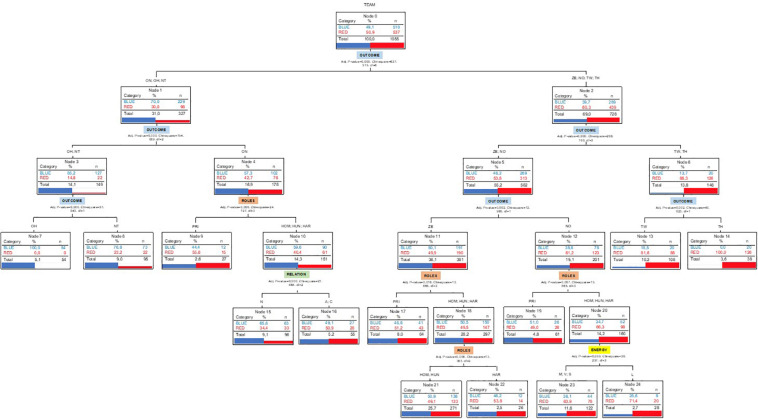
Classification tree (QUEST) to identify the predictive capacity of different variables that explain the Strategic Conduct of the Blue and Red Teams.

The roles included for the analysis were Home, Prisoner, Hunter, and Hare. The last two roles were associated with great variability in decision options, relationship, and level of physical effort. The proportion of cases correctly classified was 64.1%, the estimated risk of misclassification (0.359), and the standard error of classification (0.015) were calculated.

At all levels of the tree, some variables of the internal logic of the game appeared, such as the outcome (in the first three) and the role (in the last three). In addition, in the first three predictive levels, the outcome was the first explanatory variable.

In the fourth level, in addition to the Role, the variable Relationship was found. At the last level, Role and Energy variables emerged again. The risk type variable in the decision was not found as an explanatory variable of the conduct of the two teams. Most of the data (69%) corresponded to the prediction on the outcome ZE (0), no (–1), TW (+2), and TH (+3) (node 2).

#### Draw Outcome (ZE; Node 11 and Following)

The predictive variables were associated with the Role, which distinguished the intervention in Prisoner (node 17) and the role of Hare (node 22), in both cases more present in the red team than in the blue team. In contrast, the Hunter role was slightly higher in the blue team (node 22).

#### Unfavorable Outcomes

With the NO outcome (–1; node 12 and following). It appeared as a novelty that at Home, Hunter, and Hare roles (node 20) predicted the type of physical involvement of the teams (nodes 23 and 24), which distinguished light (node 23) from the rest of intensities of effort (node 24). In these cases, the red team was superior to the blue one. With the rest of unfavorable outcome (score), no more predictive variables were identified.

#### Favorable Outcomes

With the ON outcome (+ 1, next node 4) the same prediction of the role (distinction of the role of Prisoner from the rest) was originated to identify the strategic conduct of both teams. However, the roles HOM, HUN, and HAR predicted the relationship variable, differentiating the neutral relationship (node 15, higher in the blue team) from the adversary and partner relationship (node 16, slightly higher in the red team). With the rest of the favorable outcomes (scores), no more predictive variables were identified.

### Results: Study 2

The results shown below respond to three complementary approaches. In the first two sections, the number of T-patterns found exceeded the proposed randomization, and this approach would be validated. Free interaction between Hunters and Hares as a whole during the game (section “Strategic Temporal Regularities in the Hunter and Hare Roles in the Marro Game”) was between Hunters and Hares of both teams (section “Interaction Between the Hunters of a Team and the Hares of the Rival Team”) and finally, the strategic forms between Hunters and Hares in their own team (section “Areas of Multimodal Strategic Chains”). This specificity approach, by studying the most decisive roles within the dynamics of the game, is unprecedented.

#### Strategic Temporal Regularities in the Hunter and Hare Roles in the Marro Game

**FIGURE 5:**
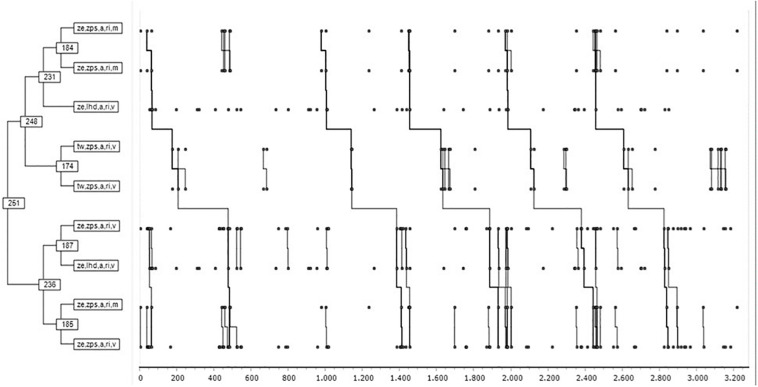
Dendrogram of temporal regularities between Hunters and Hares.

Figure 5 shows the roles of Hunter and Hare while playing. Two large groups of strategic chains were observed:

(a) **Draw outcome and superiority, two points favorable**
  a.1 Draw outcome. The first subgroup corresponded to the draw outcome. Two chains were identified (ze, zps, a, ri, m; ze, zps, a, ri, m), after that double sequence it was followed (ze, lhd, a, ri, v).
  a.2 With favorable outcome (+ 2): (tw, zps, a, ri, v tw, zps, a, ri, v).
(b) **Draw outcome (score)**
  b.1 The first subgroup was composed of two strategic chains (ze, zps, a, ri, v ze, lhd, a, ri, v).
  b.2 The second subgroup was composed of two chains. The first was made up of two strategic chains (ze, zps, a, ri, m; ze, zps, a, ri, v): the sequence of strategic regularities 360°could be expressed as follows: ((((ze,zps,a,ri,m ze,zps,a,ri,m) ze,lhd,a,ri,v)(tw,zps,a,ri,v))((ze,zps,a,ri,v ze,lhd,a,ri,v)(ze,zps,a,ri,m ze,zps,a,ri,v))).

#### Interaction Between the Hunters of a Team and the Hares of the Rival Team

**FIGURE 6:**
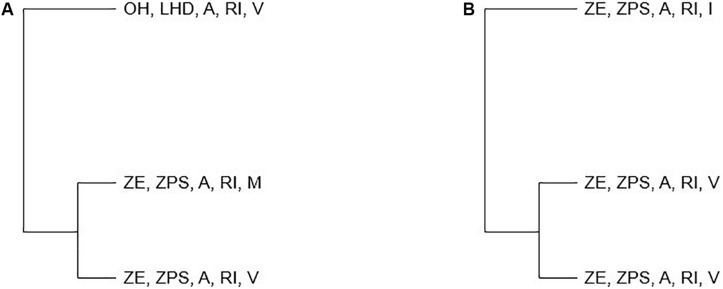
**(A)** left; (Red Hunters-Blue Hares) and **(B)** right; (Blue Hunters-Red Hares). Dendrogram of the temporal regularities the interaction between the hunters of a team and the hares of the rival team.

This section considers the interaction of the Red Hunters with the Blue Hares (Figure 6A) and of the Blue Hunters with the Red Hares (Figure 6B).

In general, in both dendrograms, there was some regularity in the use of ze, zps, a, ri, v (Draw-Chasing-Risky-Rival-Vigorous), but each team offered its own particularities. Specifically, to the left of the image, an unfavorable outcome in three units belonged to blue team with Hares trying to escape from adversary (Chain C1). This first chain was associated with the red team through two chains related to the Hunter in pursuit actions in a draw, and in moderate effort (C2) and vigorous (C3). In turn, the strategic chain (C2) and chain (C3) were closely linked.

On the other hand, the right side of the image revealed less variability than the previous dendrogram, given that only “Energy” presented changes. Thus, the strategic chain (ze, zps, a, ri) invariably appeared in the strategic sets, while the intensity of the effort changed between (C1) and (C2) from light to vigorous, respectively.

#### Areas of Multimodal Strategic Chains

**FIGURE 7:**
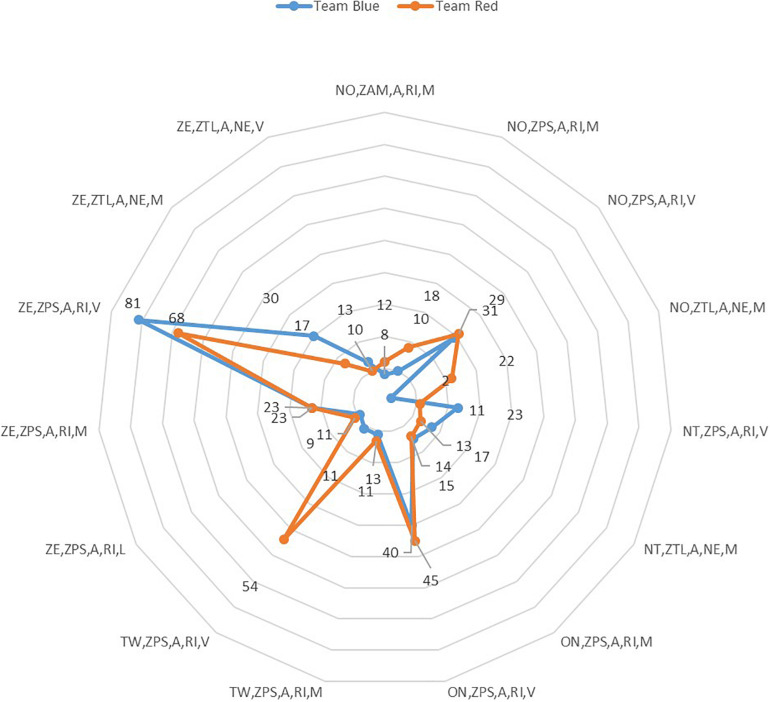
Marro360° strategic chains used by the two teams in the role of Hunter.

In addition to the analyses performed, Figures 6, 7 were constructed to identify the frequency areas of 360° multimodal strategic chains of each team in the Hunter and Hare roles.

Figure 7 shows the strategic chains most used by the teams (=2% intra-role: Hunter) in the game of Marro360°.

The Marro360°chain (ze, zps, a, ri, v) was the most used by the Hunters of both teams. Although the blue team surpassed (*n* = 81) the red team (*n* = 61), however, while the blue team obtained the second position (*n* = 45) (on, zps, a, ri, v), it was instead the strategic set (*n* = 54) (tw, zps, a, ri, v) achieved by the red team in the same second position. Within this last strategic set, the blue team was inferior (*n* = 11). Contrary to what happened with the Hare role, it was not possible to verify the exclusivity toward one of the teams, that is, both obtained some frequency.

However, the most used strategic chain in the second position by the blue team was registered with the On outcome (+ 1, *n* = 45) (on, zps, a, ri, v), while for the red team its second chain was identified with the outcome Tw (+ 2, n = 54, tw, zps, a, ri, v).

In general, regarding the differences between both teams, it could be seen that the red team was more active in the Hunter role (*n* = 40). These differences were found primarily in five sets equal to or greater than 12 units. While three sets of variables were found in the blue team (*n* = 12; nt, zps, a, ri, v) (*n* = 13; ze, zps, a, ri, v) (*n* = 13; ze, ztl, a, ne, m), two sets were identified in the red team (*n* = 20; no, ztl, a, ne, m) and (*n* = 43; tw, zps, a, ri, v).

**FIGURE 8:**
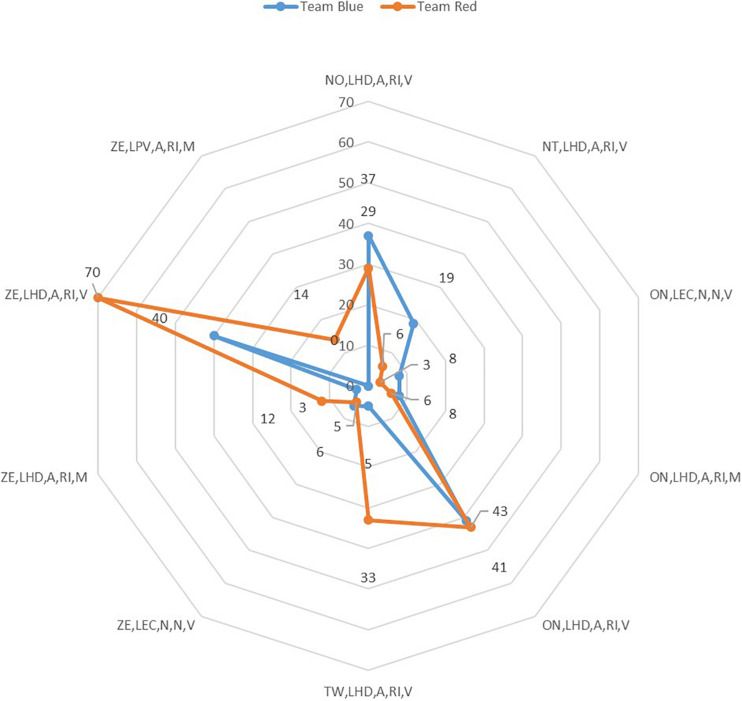
Marro360° strategic chains used by the two teams in the role of Hare.

Figure 8 shows the strategic forms of both teams. The sets of variables (frequencies) most used by the teams (=2% intra-role: Hare).

The more characterizing Marro360° strategic chains differed by teams. While the blue team (*n* = 41) used the first position (on, lhd, a, ri, v), in the red team it occupied the second position (*n* = 43). These few differences increased when comparing the two most used Marro360° strategic chains (ze, lhd, a, ri, v) by the red team (*n* = 70) against the blue team (*n* = 40). Other strategic forms Marro360° didn’t appear in the red team (*n* = 14) (ze, lpv, a, ri, m).

As in the Hunter role, the most active Hares were found in the red team (*n* = 54). When selecting the differences equal to or greater than 12 units, four sets were found, three favorable to the red team (*n* = 14; ze, lpv, a, ri, m) (*n* = 28; tw, lhd, a, ri, v) (*n* = 30; ze, lhd, a, ri, v) and one to the blue team (*n* = 13; nt, lhd, a, ri, v).

## Discussion

This research has responded to the objective of the two studies to determine the Marro360° multimodal strategic intervention (decisional, relational, and organic) of two teams that face each other in a Marro game based on the outcome and the roles of the game.

By participating in any TSG (and also in a sport), players try to respond to the problems proposed by the internal logic of that activity (Parlebas, 1988, 2001, 2017b) through motor conducts that testify to a decisional, relational, and energetic intelligence. Therefore, the effect of two independent variables from the game were studied: the score and the subroles associated with the Hunter and Hare roles on the dependent variables represented by Marro360° strategic chains. Each of these chains was simultaneously integrated by the type of response according to the decision risk (risk in the decision), the relationship, and the energy intensity (Reimers et al., 2018). It is unavoidable to mention that the methodological approach used by the present study became feasible, since the integration of different variables (dynamic score, role, subrole, relation, decision risk, energy) was addressed.

From this perspective, the study has revealed how two teams with antagonistic interests, subject to the same rules of the game, reveal a specific collective intelligence, through the analysis of individual motor responses (individually considered variables) but, above all, complex strategic chains Marro360° (decisional, relational, and energetic) capable of characterizing the participation of each team.

Knowing the internal logic of the game has led us to follow a consistent design of mixed methods (observational) (Camerino et al., 2012; Preciado et al., 2019), which has allowed expert observers to build a consensual registration system, viable in practice and reliable. Thus, the experiences of the teams have been recorded from a rigorous and ecological approach, capable of capturing the interactive spontaneity (Parlebas, 1988) of the context (Guerreiro et al., 2019). According to Marro’s internal logic, the timeline of events (Hancock and Block, 2012; Timoszyk-Tomczak and Bugajska, 2018) acquires special relevance unlike other traditional games or sports in which the temporal relationship is not so crucial to modify the outcome. The deep knowledge of the internal logic of Marro makes it possible to state with severity that the ability of teams to calculate and manage time is a distinctive feature of the motor conducts of the participants.

The Marro360° strategy of the game reaches its zenith in making decisions for the Hunter and Hare roles because they are the ones who can unbalance the scoreboard (capturing opponents or saving teammates). The study identifies the frequency of strategic chains for these roles (Figures 7, 8), as well as their temporal recurrence through dendrograms. The observation of each player, also explored in other ecological contexts of traditional and sports games (Araújo et al., 2014), has enriched the understanding of the strategic conduct of both teams.

The ambition of this study has been to address the analysis of the process of formation of Marro360° strategic chains in both teams from a multifaceted approach to the motor conducts of their players. This has been possible thanks to the use of mixed methods that have also been effective in other studies (Johnson et al., 2007; Anguera et al., 2018; Casal et al., 2019).

In a first overview of the game, the different types of statistical analyses (cross tables, classification tree, and the first dendrogram: Figure 5) show that the draw outcome (ZE) is a relevant variable of the strategic framework in the Marro game. In situations of equality on the outcome is when both teams generate greater numbers of T-patterns (Casarrubea et al., 2016, 2019b). This finding suggests a clear pedagogical transfer: teachers should teach students how to manage Marro360° strategies with the draw outcome, both at the start of the game and during the confrontation.

In this global perspective of the game, in the dendrogram, two key outcomes were observed: in a draw (ZE) and favorable outcome + 2 (TW) with the emergence of T-patterns (Casarrubea et al., 2018; Magnusson, 2020) of Marro360° in terms of temporal chains (((((ze, zps, a, ri, m ze, zps, a, ri, m) ze, lhd, a, ri, v) (tw, zps, a, ri, v tw, zps, a, ri, v)) ((ze, zps, a, ri, v ze, lhd, a, ri, v) (ze, zps, a, ri, m ze, zps, a, ri, v)))). These chains integrate the risky subroles (ri) Hunter-chasing (zps) and Hare-fleeing (lhd) of high rivalry relation directed to the adversaries (a), associated with a moderate-to-vigorous energy intensity (m-v).

This photograph of the temporal regularities of the game of Marro360°, although it did not distinguish the teams, shows the teacher the complexity of the strategic time management that emerged from the game, that is to say, the class group. However, we went further trying to interpret the Marro360° strategic intervention of both teams during the game. This challenge was specified when both teams participated in the roles of Hunter and Hare.

### Marro360° Intervention of the Red Hunters on the Blue Hares (Figure 6A)

It should be noted that, in the Marro game, the Hunter and Hare roles are essentially operational procedures (Parlebas, 2019) that are decisive for modifying the outcome, although playing these roles does not imply winning the game directly. By including the outcome variable in the analysis, through crossed tables, differences in the outcome (*p* < 0. 001) of both teams were observed when participating under Hunter and Hare roles. With the outcome on disadvantage NT (–2), both teams show an unfavorable situation, although with a different proportion: blue team (*n* = 104; AR = 7.2; 6.8%) and red team (*n* = 38; AR = –7.2; 2.5%). When the outcome was favorable TW (+2), the red team (*n* = 134; AR = 6.9; 8.8%) exceeded the blue team (*n* = 33; AR = –6.9; 2.2%). In addition, in the dendrogram two T-patterns (Magnusson, 2020) were detected when the blue team intervened before an unfavorable outcome (ON = –1, lhd = flee, a = rival, ri = risky, v = vigorous). This chain was predictive (T-prediction) and more present in the blue team than in the red one, as shown in the second dendrogram (Figure 6A) (*n* = 5, length = 3), which confirms a situational advantage (Gómez et al., 2017) for the red team. This dendrogram was relevant since it offers a greatest complexity of temporal regularities between red team Hunters and blue team Hares, with similar analyses used in previous studies (Pic et al., 2018). Although other T-patterns were found in 70% of the records (as in Figure 6A), none of the following strings in the example were predictive (*n* = 5, length = 3) [tw, zps, a, ri, v (nt, lhd, a, ri, v tw, zps, a, ri, v)].

In relation to the role of Hunter, the results show that the red team exceeds the blue in frequency of strategic Marro360°chains. For the red Hunters, the Marro360°chain that best represents them is given with the outcome in advantage for an additional 2 points (TW = + 2, zps = Hunter-chasing, a = rival, ri = risky, v = vigorous). In addition, the classification tree has shown that the TW (+2) outcome was a key variable to predict the difference in the strategic behavior of both teams (node 13, red: *n* = 88; blue: *n* = 20).

On the other hand, despite the constant variability of the outcome in this game, the second-most frequent Marro360°chain of the red team was identified with an unfavorable outcome (NO = –1, ztl = Hunter-transfer, a = rival, ne = neutral, m = moderate).

Therefore, although it can be affirmed that, in general, in the role of Hunter the red team was superior to the blue one, it is also true that, in some sections of the game, the blue team acquired superiority over its rival. This test confirms once again the originality of this study against other precedents, when trying to reveal the process of the game, that is, the analysis of the strategic use of time in the sociomotor dynamics that originate the plays in the Marro game.

### Marro360° Intervention of the Blue Hunters on the Red Hares (Figure 6B)

The third dendrogram (Figure 6B) showed little variability when blue Hunters faced red Hares. Marro360°chains appeared with the tie outcome (ZE), and only the energy intensity variable (light or vigorous) was modified [ze, zps, a, ri, l (ze, zps, a, ri, v ze, zps, a, ri, v)].

The interpretation of this finding may lead to several reflections. First, it is likely that from the moment the blue team Hunters vary their outcome they change the way they act and, therefore, no other combinations or outcome were observed. Sometimes, players start the game little concentrated or, facing too much novelty, they are less active as shown by the intensity of light energy. However, they become active quickly (vigorously) to get to hunt rival Hares.

As to why no T-patterns have been found between the red Hares and the blue Hunters, as was the case with the analogous dendrogram (Figure 6A), although with great caution, we are inclined to consider that surely these Hares offered more variety of responses than the answers offered by blue Hares since, if the blue team Hunters have been temporarily regular, some temporary regularity should also have been revealed. On the other hand, it could also be due to the great predominance of the blue team in Hunters, since a search was made of all the T-patterns (length = 3), and only the participation of the red team (Hares) was found in a dendrogram of all six found.

This finding suggests a direct transfer to the educational field: the educator should educate toward the coding and decoding of strategic messages. That is, teachers should stimulate in their students the creation (coding) of varied, unpredictable strategic messages that hinder the detection of Marro360°chains. In parallel, students should be encouraged to detect (decode) temporary regularities in the strategic action plan of the opposing team.

An unexpected finding in this game has been the non-detection of Marro360°chains aimed at saving partners, even though the Marro game allows one to gain an advantage on the outcome by saving teammates or capturing opponents. It has been observed that both teams used primarily regularities with opposition relations (capture) in the face of intra-team cooperation. The reason may be due to a poor strategic approach. It could be thought that the majority of the participants have a “sports footprint” as a result of their background in team sports in which victory is achieved through motor relations projected exclusively on the opponent (insert the ball into the goal or rival zone).

The theoretical procedures and the methodological design of mixed methods employed have allowed us to identify the strategic intervention of both teams, as well as finding T-patterns (Magnusson, 2000) based on outcome. This multifaceted vision allows an interpretation of the process followed during a game in the confrontation of two teams. The two groups were different because their 360° strategic chains were uneven. The risk in the subrole, the type of relationship, and the intensity of energy used have been different in both teams. Situational variables (Gómez et al., 2017) asks for managing time as a faithful ally and focusing on the dynamics of the interactive collective process (Gonçalves et al., 2016; Parlebas, 2019) of the intervening teams.

It has been observed that the red team was more active than the blue team in the roles of Hunter and Hare, which could explain its superiority. However, that reading should be done with caution, since, being a game with such a variable outcome, the momentary advantage could be changed to an unfavorable situation. It seems reasonable to interpret that when the Hare of any team intensified its activity, it was due to an emergency not chosen in that situation, in which the rival team had a domain or advantage over the subsequent options in the Marro game.

Deeply understanding the keys of the Marro game without referring to its temporal dimension would be a complex task, provided that the interactive process is considered relevant for the study of the game. Leaving Home “after” an adversary grants a strategic advantage. In addition, the time to make decisions is reduced, and the decision options are numerous and changing (Parlebas, 2010). Thus, choosing the right time to change roles (leaving Home, moving from Hunter to Hare, deciding whether to chase or flee, save or capture) is a key aspect of multimodal strategic intervention. There is scientific evidence that shows that Marro’s game relationships go from binary strategic relationships (Parlebas, 2019) in players aged 7–8 to relationships that increase in complexity (Morin, 1990) in adults. While a player is chasing an adversary (binary relationship), he/she is being chased by another rival (tertiary relationship), which in turn can be harassed by a partner (quaternary relationship).

Among the most notable limitations, the increase in participants would be advisable to know the aprioristic strategic plans of the players before starting the game, and their subsequent comparison would be a future line of suggestive exploration by research groups, as well as the used of test-posttest designs to find out specific T-patterns for each player. On the other hand, studying the emotional state of the players, as well as the personality traits of the participants, would offer great opportunities to get to know the player better and thus establish better curricular plans in the future. In this study, the scarcity of specific literature used mostly in discussion, responded to the small group of papers in line with the objectives of the study. Therefore, it was complex to establish direct comparisons with other groups and research studies.

The study reveals direct transfers in the educational field. The traditional game in general, and the Marro game in particular, offer an extraordinary setting to develop fundamental learning of physical education (Parlebas, 2017a, 2019). Educating strategies based on the outcome, especially when teams draw, trying to identify in the opposing team temporary T-patterns of multimodal 360°chains, as well as avoiding being predictable for rivals, are some pedagogical examples that emerge based on this experience.

## Conclusion

The most relevant strategic chains in the Marro game have been specified from different variables and approaches. It is a game that made driving conducts of decision, relationship (Heider, 1946) (positive and negative valences), and physical effort emerge, which activate multimodal learning of motor skills in its participants (Parlebas, 2017a; Ward et al., 2017). Through Marro the participants developed values of coexistence necessary for current society, recognized by different official international initiatives of UNESCO (e.g., Kazan 2017 action plan, 2030 Agenda, Berlin Declaration, and the International Charter of EF, AF, and Sport).

This study has deepened on a 360° multimodal vision, rather unknown in studies around TSGs and sports. The variables and strategic forms (multimodal chains of Marro360° variables) used by two teams depending on the outcome, aligned with the temporal transience (T-patterns) and the sociomotor dynamics of the process rather than the final result, revealed the educational potential of this game (Parlebas, 2019). The internal logic of the Marro game asks the protagonists to intervene in a systemic (multimodal) way, activating at the same time the decision, the relationship, and the intensity of energy according to their strategic interests (Lavega et al., 2016).

The analysis made by using the classification tree confirmed the great relevance of the outcome and role variables and, to a lesser extent, energy and relation, as predictive factors of the conduct in both teams. More specifically, the Hunter and Hare roles were decisive for the study of Marro, since the dominance of the red team over the blue team nested on favorable outcomes, but also on the role, with greater exposure and development of the most decisive roles. This suspected predominance was intensified when the Hunters in the blue team and the Hares in the red team generated a dendrogram with a more favorable outcome (score) for the team of the Hares (belonging to the red team). However, in the homologous dendrogram, no T-patterns were identified for Hares in the red team (Figure 6B). This finding could be related to the high decision variability of the role of Hare in the red team, or due to the scarcity of actions by Hunters belonging to the blue team and/or their limited strategy. Therefore, playing is not neutral. The physical education teacher has evidence on (multimodal) development in the game of Marro360°. Knowing hosted information about players and strategic roles (decisional, relational, and organic) can help teachers make better decisions in their daily tasks.

## Data Availability Statement

The datasets generated for this study are available on request to the corresponding authors.

## Ethics Statement

The studies involving human participants were reviewed and approved by the Ethics Committee for Clinical Research of the Catalan Sports Council. Generalitat de Catalunya. The patients/participants provided their written informed consent to participate in this study.

## Author Contributions

PL-B, CS-S, JS, MP, and AE: substantial contribution to study conception and design. PL-B, US, AR-A, VM-A, and QP: preparation of the document for approval by the ethics committee. PL-B, QP, VM-A, SD-S, AE, LM, PA-A, and CS-S: preparation and participation in the empirical work. RL-P, CS-S, PA-A, RR-A, and JS: observational analysis, manual observational preparation, strategic chain analysis of the players. AE, LM, and DM-M: download and analysis of all accelerometry data. MP, VM-A, AE, RL-P, CS-S, JS, and PL-B: preparation of the temporary database (all variables). MP, PL-B, VM-A, SD-S, JS, RL-P, AR-A, DM-M, and PA-A: database revision. MP, PL-B, DM-M, RR-A, US, PA-A, JS, RL-P, CS-S, and AE: discussion of data analysis strategies. PL-B, MP, AE, LM, US, AR-A, RR-A, CS-S, JS, DM-M, VM-A, and QP: writing of the manuscript. All authors contributed to the article and approved the submitted version.

## Conflict of Interest

The authors declare that the research was conducted in the absence of any commercial or financial relationships that could be construed as a potential conflict of interest.

## References

[B1] Aiello S. Crescimanno G. Di Giovanni G. Casarrubea M. (2020). T-patterns in the study of movement and behavioral disorders. *Physiol. Behav.* 215:112790. 10.1016/j.physbeh.2019.112790 31870941

[B2] Anguera M. T. Blanco A. Hernández-Mendo A. Losada J. L. (2011). Observational designs: their suitability and application in sports psychology. *Cuad. Psic. Dep.* 11 63–76.

[B3] Anguera M. T. Camerino O. Castañer M. Sáchez-Algarra P. (2014). Mixed methods in physical activity and sport. *Rev. Psicol. Dep.* 23 123–130.

[B4] Anguera M. T. Blanco-Villaseñor A. Losada J. L. Sánchez-Algarra P. Onwuegbuzie A. J. (2018). Revisiting the difference between mixed methods and multimethods: is it all in the name? *Qual. Quant.* 52 2757–2770. 10.1007/s11135-018-0700-2

[B5] Apostolidis N. Nassis G. P. Bolatoglou T. Geladas N. D. (2003). Physiological and technical characteristics of elite young basketball players. *J. Sport. Med. Phys. Fi.* 44 157–163. 15470313

[B6] Araújo D. Silva P. Ramos J. P. (2014). Affordance-based decisions guide team synergies during match performance. *Res. Phys. Edu. Sport. Health.* 3 19–26.

[B7] Ato M. López J. J. Benavente A. (2013). A classification system for research designs in psychology. *Anal. Psicol. Spain* 29 1038–1059. 10.6018/analesps.29.3.178511

[B8] Bangsbo J. (2000). “Physiology of intermittent exercise,” in *Exercise and Sport Science*, eds Garrett W. E. Kirkendall D. T. (Philadelphia: Lippincott Williams & Wilkins), 53–65.

[B9] Berge C. (1958). *Theory of Graphs and its Applications.* París: Dunod.

[B10] Blanco-Villaseñor A. Castellano J. Hernández-Mendo A. Sánchez-López C. R. Usabiaga O. (2014). Application of GT in sport for the study of the reliability, validity and estimation of the sample. *Rev. Psicol. Deporte* 23 131–137.

[B11] Böhm R. Rusch H. Baron J. (2018). The psychology of intergroup conflict: a review of theories and measures. *J. Econ. Behav. Organ.* 10.1016/j.jebo.2018.01.020

[B12] Brasó J. Torrebadella X. (2017). The game prisoner’s bar: a research on their educational backgrounds. *Rev. Dialect. Trad. Pop.* 72 245–264. 10.3989/rdtp.2017.01.010

[B13] Bredemeier B. (1991). “Morality and sport for all,” in *Sport for All*, eds Oja P. Telama R. (Amsterdam: Elsevier), 365–372.

[B14] Brill M. Schwab F. (2019). A mixed-methods approach using self-report, observational time series data, and content analysis for process analysis of a media reception phenomenon. *Front. Psychol.* 10:1666. 10.3389/fpsyg.2019.01666 31396130 PMC6667654

[B15] Camerino C. Castañer M. Anguera M. T. (2012). *Mixed Methods Research in the Movement Sciences: Cases in Sport, Physical Education and dance.* New York, NY: Routledge.

[B16] Cardinet J. Johnson S. Pini G. (2010). *Applying Generalizability Theory using EduG.* New York, NY: Routledge.

[B17] Casal C. A. Anguera M. T. Maneiro R. Losada J. L. (2019). Possession in football: more than a quantitative aspect–a mixed method study. *Front. Psychol.* 10:501. 10.3389/fpsyg.2019.00501 30936844 PMC6431675

[B18] Casarrubea M. Aiello S. Santangelo A. Di Giovanni G. Crescimanno G. (2019a). Different Representation procedures originated from multivariate temporal pattern analysis of the behavioral response to pain in wistar rats tested in a hot-plate under morphine. *Brain Sci.* 9:233. 10.3390/brainsci9090233 31547468 PMC6770233

[B19] Casarrubea M. Aiello S. Di Giovanni G. Santangelo A. Palacino M. Crescimanno G. (2019b). Combining quantitative and qualitative data in the study of feeding behavior in male wistar rats. *Front. Psychol.* 10:881. 10.3389/fpsyg.2019.00881 31068869 PMC6491709

[B20] Casarrubea M. Faulisi F. Caternicchia F. Santangelo A. Di Giovanni G. Benigno A. (2016). Temporal patterns of rat behaviour in the central platform of the elevated plus maze. Comparative analysis between male subjects of strains with different basal levels of emotionality. *J. Neurosci. Methods* 268 155–162. 10.1016/j.jneumeth.2015.07.024 26247889

[B21] Casarrubea M. Jonsson G. K. Faulisi F. Sorbera F. Di Giovanni G. Benigno A. (2015). T-pattern analysis for the study of temporal structure of animal and human behaviour: a comprehensive review. *J. Neurosci. Meth.* 239 34–46. 10.1016/j.jneumeth.2014.09.024 25280983

[B22] Casarrubea M. Magnusson M. Anguera M. T. Jonsson G. K. Castañer M. Santangelo A. (2018). T-pattern detection and analysis for the discovery of hidden features of behaviour. *J. Neurosci. Methods.* 310 24–32. 10.1016/j.jneumeth.2018.06.013 29935197

[B23] Chacón-Moscoso S. Anguera M. T. Sanduvete-Chaves S. Losada J. L. Lozano-Lozano J. A. Portell M. (2019). Methodological quality checklist for studies based on observational methodology (MQCOM). *Psicothema.* 31 458–464. 10.7334/psicothema2019.116 31634092

[B24] Cohen J. (1988). *Statistical Power Analysis for the Behavioural Sciences.* Hillsdale, NJ: Erlbaum.

[B25] Cronbach L. J. Gleser G. C. Nanda H. Rajaratnam N. (1972). *The dependability of Behavioral Measurements: Theory of Generalizability for Scores and Profiles.* New York, NY: John Wiley and Sons.

[B26] Gibbons S. Ebbeck V. Maureen R. (1995). Fair play for kids: effects on the morale development of children in physical education. *Res. Q. Exerc. Sport* 66 247–255. 10.1080/02701367.1995.10608839 7481086

[B27] Gómez M. Á Rivas F. Connor J. D. Leicht A. S. (2019). Performance differences of temporal parameters and point outcome between elite men’s and women’s badminton players according to match-related contexts. *Int. J. Env. Res. Pub.* 16:4057. 10.3390/ijerph16214057 31652686 PMC6862575

[B28] Gómez M. Á Silva R. Lorenzo A. Kreivyte R. Sampaio J. (2017). Exploring the effects of substituting basketball players in high-level teams. *J. Sport. Sci.* 35 247–254. 10.1080/02640414.2016.1161217 26986448

[B29] Gonçalves B. Marcelino R. Torres-Ronda L. Torrents C. Sampaio J. (2016). Effects of emphasising opposition and cooperation on collective movement behaviour during football small-sided games. *J. Sport. Sci.* 34 1346–1354. 10.1080/02640414.2016.1143111 26928336

[B30] Graupensperger S. Panza M. Evans M. B. (2019). Network centrality, group density, and strength of social identification in college club sport teams. *Group. Dyn Theor. Res.* 24 59–73. 10.1037/gdn0000106 PMC7453997 32863704

[B31] Guerreiro C. F. Harvey S. Hastie P. A. Mesquita I. M. R. (2019). Effects of situational constraints on students’ game-play development over three consecutive Sport Education seasons of invasion games. *Phys. Educ. Sport. Peda.* 24 267–286. 10.1080/17408989.2019.1571184

[B32] Hancock P. A. Block R. A. (2012). The psychology of time: a view backward and forward. *Am. J. Psychol.* 125 267–274. 10.5406/amerjpsyc.125.3.0267 22953687

[B33] Heider F. (1946). Attitudes and cognitive organization. *J. Psychol.* 21 107–112. 10.1080/00223980.1946.9917275 21010780

[B34] Hernández-Mendo A. Blanco-Villaseñor A. Pastrana J. L. Morales-Sánchez V. Ramos-Pérez F. J. (2016). SAGT: computer application for generalizability analysis. *Rev. I. Psicol. Ejerc, el Deporte* 11 77–89.

[B35] Hernández-Mendo A. Planchuelo-Medina L. (2012). An observational tool for the evaluation of moral development in primary school physical education classes. *Rev. I. Psicol. Ejercic. el Deporte* 7 287–306.

[B36] IBM SPSS Statistics, v. 25 (2017). *International Business Machines Corp.* Armonk, NY: IBM.

[B37] Johnson R. B. Onwuegbuzie A. J. Turner L. A. (2007). Toward a definition of mixed methods research. *J. Mix. Method. Res.* 1 112–133. 10.1177/1558689806298224

[B38] Lavega P. Alonso J. I. Etxebeste J. Lagardera F. March J. (2014a). Relationship between traditional games and the intensity of emotions experienced by participants. *Res. Q. Exercise. Sport.* 85 457–467. 10.1080/02701367.2014.961048 25412128

[B39] Lavega P. Planas A. Ruiz P. (2014b). Cooperative games and inclusion in physical education. *Rev. Int. Med. Cienc. Act.* 14 37–51.

[B40] Lavega P. Etxebeste J. Sáez de Ocariz U. Serna J. During B. (2016). “Apprendre à vivre ensemble par les Jeux sportifs traditionnels,” in *Égalité, mixité, intégration par le sport*, ed. Ferréol G. (Paris: Éditions l’Harmattan), 129–146.

[B41] Lee M. (1988). Values and responsibilities in children’s sports. *Phys. Educ. Rev.* 11 19–27.

[B42] Levi-Strauss C. (1963). *Structural Anthropology. Translated by Claire Jacobson and Brooke Grundfest Schoepf.* New York, NY: Basic Books.

[B43] Magnusson M. (2020). T-Pattern detection and analysis (TPA) with THEME: mixed methods approach. *Front. Psychol.* 10:2663. 10.3389/fpsyg.2019.02663 31998165 PMC6965347

[B44] Magnusson M. S. (2000). Discovering hidden time patterns in behaviour: T-patterns and their detection. *Behav. Res. Meth. Ins. C.* 32 93–110. 10.3758/BF03200792 10758668

[B45] Morin E. (1990). *Introduction to Complex Thought.* Madrid: Gedisa.

[B46] Muñoz V. Lavega P. Serna J. Sáez U. March J. (2017). Mood states when playing alone or in cooperation: two unequal motor and affective experiences. *An. Psicol.* 33 196–203. 10.6018/analesps.33.1.233301

[B47] Navarro V. Triguero C. (2009). *Research and motor game in Spain.* Lleida: Universitat Lleida.

[B48] Parlebas P. (1988). *Elements of Sociology in Sport.* Málaga: Unisport.

[B49] Parlebas P. (2001). *Contribution to an Annotated Vocabulary on the Science of Motor Action.* Barcelona: Paidotribo.

[B50] Parlebas P. (2002). Réseaux dans les jeux et les sports [Networks in games and sports]. *L’Année Socio.* 2 314–349. 10.3917/anso.022.0314 18052372

[B51] Parlebas P. (2005). “The game, emblem of a culture,” in *Enciclopedia Catalana Jocs i Esports tradicionals, Tradicionari, Enciclopèdia de la cultura popular de Catalunya: 17* (Barcelona: Enciclopedia catalana).

[B52] Parlebas P. (2010). “Health and relationship wellness in traditional games,” in *Jeux Traditionnels et Santé Sociale*, ed. De La Villa C. (Aranda de Duero: Asociación cultural la Tanguilla), 95–101.

[B53] Parlebas P. (2017a). A pedagogy of motor skills. *Acción Motriz* 20 89–95.

[B54] Parlebas P. (2017b). *La aventura praxiológica. Cencia, acción y educación física.* Málaga: Junta de Andalucía.

[B55] Parlebas P. (2019). Jeux traditionnels. *Vers l’Educ. Nouvelle* 575 46–53.

[B56] Pic M. Lavega-Burgués P. March-Llanes J. (2019). Motor behaviour through traditional games. *Educ. Stud.* 45 742–755. 10.1080/03055698.2018.1516630

[B57] Pic M. Navarro-Adelantado V. Jonsson G. K. (2018). Detection of ludic patterns in two triadic motor games and differences in decision complexity. *Front. Psychol.* 8:2259. 10.3389/fpsyg.2017.02259 29354084 PMC5760529

[B58] Preciado M. Anguera M. T. Olarte M. Lapresa D. (2019). Observational studies in male elite football: a systematic mixed study review. *Front. Psychol.* 10:2077. 10.3389/fpsyg.2019.02077 31681054 PMC6813914

[B59] Reimers A. K. Schoeppe S. Demetriou Y. Knapp G. (2018). Physical activity and outdoor play of children in public playgrounds-do gender and social environment matter? *Int. J. Environ. Res. Public. Health.* 15 1–14. 10.3390/ijerph15071356 29958386 PMC6069007

[B60] Rousseau J.-J. (1973). in *The Social Contract and Discourses*, ed. Cole G. D. J. (London: Dent Dutton).

[B61] Sackett G. P. (1978). *Observing Behavior. Vol. II: Data Collection and Analysis Methods.* Baltimore, MD: University Park Press.

[B62] Teddie C. Tashakkori A. (2010). “Overview of contemporary issues in mixed methods research,” in *The Sage Handbook of Mixed Methods in Social & Behavioral Research*, 2nd Edn, eds Tashakkori A. Teddie C. (Thousand Oaks: Sage), 1–41.

[B63] Theme (2017). *Version 6) [Computer software].* Reykjavík: Pattern Vision Ltd.

[B64] Timoszyk-Tomczak C. Bugajska B. (2018). Transcendent and transcendental time perspective inventory. *Front Psychol.* 10:2677. 10.3389/fpsyg.2018.02677 30687156 PMC6335272

[B65] Troiano R. P. Berrigan D. Dodd K. W. Masse L. C. Tilert T. McDowell M. (2008). Physical activity in the United States measured by accelerometer. *Med. Sci. Sport. Exer*. 40 181–188. 10.1249/mss.0b013e31815a51b3 18091006

[B66] UNESCO (2017). *Conferencia Internacional de Ministros y Altos Funcionarios Encargados de la Educación Física. SHS/2017/PI/H/14 REV.* Kazan: UNESCO.

[B67] Ward N. Paul E. Watson P. Cooke G. E. Hillman C. H. Cohen N. J. (2017). Enhanced learning through multimodal training: evidence from a comprehensive cognitive, physical fitness, and neuroscience intervention. *Sci. Rep.U. K.* 7 5808. 10.1038/s41598-017-06237-5 28724914 PMC5517605

[B68] Ysewijn P. (1996). *About Software for Generalizability Studies (GT).* Switzerland: Mimeograph.

